# Notes of a Visit to Some of the Asylums of Great Britain

**Published:** 1883-11

**Authors:** Edward N. Brush

**Affiliations:** Assistant Physician, New York State Lunatic Asylum


### Selections.

### NOTES OF A VISIT TO SOME OF THE ASYLUMS OF GREAT BRITAIN.

By EDWARD N. BRUSH, M.D., Assistant Physician, New York State Lunatic Asylum.

The opening of a short summer vacation found me in England, and it was with many anticipations of profit and pleasure that I resolved upon observing, as far as the time at my disposal would allow, something of the English methods of lunacy administration.

The institutions visited, and upon which the following notes are based, are named below in the order seen :

| Name. | Superintendent. | No. of Patients. |
|---|---|---|
| Lancashire Lunatic Asylum, Lancashire Moor, | Dr. David Cassidy, | 1,140 |
| Royal Albert Asylum for Idiots, Lancaster, | Dr. G. E. Shuttleworth, | 600 |
| Royal Edinburgh Asylum for Insane, | Dr. T. S. Clouston, | 830 |
| Barony Parochial Asylum for Insane, Lenzie, Scotland, | Dr. James Rutherford, | 476 |
| Glasgow Royal Asylum for Insane, Gartnavel, | Dr. David Yellowlees, | 500 |
| West Riding Lunatic Asylum, Wakefield, | Dr. Herbert C. Major, | 1,400 |
| Bethlem Royal Hospital for Lunatics. London, | Dr. Geo. H. Savage, | 245 |
| Middlesex County Lunatic Asylum, Colney Hatch, | Dr. Edgar Sheppard, Mr. W. G. Marshall, | 2,200 |
| Middlesex County Lunatic Asylum, Hanwell, | Dr. Henry M. Rayner, | 1,800 |
| Surrey County Lunatic Asylum, Brookwood, | Mr. James E. Barton, | 1,100 |
| Lancashire Lunatic Asylum, Prestwich, | Mr. H. Rooke Ley, | 1,200 |

With but one trifling exception I was cordially received, and my questions were pleasantly answered upon all topics connected with asylum management and the treatment of the insane.

Though visiting Great Britain at the time of the usual summer vacation, I had the good fortune to find all but one of the medical superintendents at home. Dr. Clouston, of Morningside, unhappily for me, was absent for his yearly holiday.

Lancashire, where my first experience in English Asylums was gained, has a population of about three and a quarter millions, with 7,500 lunatics, while adequate asylum accommodation was, prior to the annexes, at Lancaster, Prestwich, Rainhill and Whittingham, provided for but about 4,000. The remainder are detained in workhouses or in the families of patients.

At the old asylum on Lancaster Moor over eleven hundred patients are excellently cared for under the superintendence of Dr. Cassidy.

The building, which is one of the oldest now in use for asylum purposes in England, hardly meets the modern ideas of hospital and asylum construction. The corridors are somewhat narrow and low, and the rooms are poorly lighted—some, indeed, depending solely upon the small transom over the door. Whereever possible, however, the superintendent has introduced light and color, and many of the sitting or day rooms appear cheerful and home-like.

In this asylum I saw some experiments in progress upon the effects of colored light upon the insane. One patient was in bed in an atmosphere of deep blue. I did not learn, what if any, results had been attained. I saw no restraint, nor did I see any patient who appeared in any way to need it. The wards were quiet to a marked degree, the only noise I heard coming from the airing court for refractory men. Curiously, the most troublesome patient in the house was an American sailor, for whom mechanical restraint had been necessary upon more than one occasion. Dr. Cassidy showed a museum or collection of restraining apparatus, which his predecessor and himself had carefully preserved, instead of doing as did an enthusiastic American superintendent, making a bonfire thereof. Perhaps this is only an illustration of the difference in mental constitution between the two nations, the one conservative and considerate of the future, the other impulsive, radical and ready to make everything subordinate to a present opportunity to display social pyrotechnics.

Some of this apparatus was most ingeniously contrived, and when applied must have been "mechanical restraint" indeed. I thought when looking it over, it is not surprising that a reform in favor of non-restraint found ready welcome in England, if these appliances were in use.

Of the 1,140 patients accommodated at this asylum, but 226 sleep in single rooms. The domitories are large and did not appear overcrowded. Night attendance is provided by twelve attendants, six for each sex. The suicidal and epileptic patients are under continuous night supervision, having two attendants for each sex, who have charge of the large domitories where these patients are congregated. The faithfulness of the night attendants is tested both by inspection and by Dent's recording clocks. At the time of my visit to Lancaster, the large annex, practically another asylum, commended in October, 1879, was approaching completion. It is built of red or brown sand stone quarried under the foundation, and will accommodate 700 patients, an equal number of each sex. I was informed that the cost of this building with forty-two acres of land, new lodge, laundry and mortuary, furnishing and the laying out of roads and grounds, would be about £110,000. The plan includes a large central administration building with kitchen and a dining hall 120 by 77 feet. A corridor 780 feet in length and 12 feet wide runs through the entire building, and on each side of the central building, at right angles to the corridor, are two large wings or pavilions. In this annex single rooms are provided for but forty-eight patients of each sex.

In the top story of the central building pleasant sleeping rooms are provided for the day attendants, it being intended to provide a night staff who shall have the whole responsibility of the night duty. By this provision Dr. Cassidy expects to have his day attendants enter upon their work in a fresh condition and better prepared to discharge their trying duties with the necessary evenness of temper.

The lavatories, baths, water closets and slop sinks, are placed in small detached blocks with covered connecting corridors.

Under Dr. Cassidy's guidance I visited the Royal Albert Asylum for Idiots, which is situated a short distance from the Lunatic Asylum. This Asylum is "established for the care, education and training of idiots and imbeciles of all classes belonging to the seven northern counties, viz.: Lancashire, Yorkshire, Cheshire, Westmoreland, Cumberland, Durham and Northumberland." By the census of 1871, there were of these two classes 29,452 in England and Wales, 8,104 of whom belonged in the seven counties above named. Of these last, 2,018 were between the ages of five and twenty. The asylum is under the able superintendence of Dr. G. E. Shuttleworth, and is largely supported by contributions and the income from legacies. The patients admitted to this asylum are of two classes, viz.: Free patients between the ages of six and fifteen whose friends are proved unable to meet the lowest payment; these are elected by vote of the subscribers and received for seven years, and paying patients, who are received at any time. The charges vary according to the requirements of the case and the circumstances of the friends. Unfortunately, at the time of my visit many of the pupils were away on their summer holiday, but I was able to see practical examples of the methods employed in the institution. At meals, with a very few exceptions, the inmates gather in a general dining room, where they sit under the supervision of teachers and attendants, being divided into groups in accordance with their various conditions. The school and work rooms are bright and cheerful, and the sleeping apartments everything that could be desired. Arrangements are made for such efficient night supervision that the accident which not long since happened in a similar institution in our own State, could not, I think, be repeated here.

Private patients may have, by the expenditure of a sufficient sum, a bedroom and parlor of their own, with a special attendant and other arrangements to correspond.

In the schoolrooms I saw pupils of all grades of intelligence pursuing various studies, from the simplest kindergarten exercises to complicated mathematical problems. The proficiency and progress which some exhibited in drawing were quite remarkable. One young man showed with pardonable pride some very excellent conventional designs of his own, produced by the combination of geometrical figures, and flower and leaf outlines, which would do credit to an artist of much greater intellectual attainments. I examined some of his earlier attempts at drawing, and the progress exhibited spoke well for the care taken in his training.

Dr. Shuttleworth endeavors to make the education of his pupils as far as possible of a practical kind, and I saw boys acting as storekeepers, measuring out under the eye of the teacher various commodities by weight or measure to other pupils, who, for the time being, took the part of purchasers, and who were expected to calculate the price for a given number of pounds or quarts, and to check the storekeeper in any mistake of weight, or price charged. In this way I was informed these pupils were trained to be useful and trusty errand boys and messengers, so that when returning home their services could be utilized in this way among others Under the head of school attainments of patients, the following subjects are included: Speech, reading, writing, arithmetic, clock lesson, shop lesson, color, music and drill. Of industrial occupations the following are reported: Tailoring, shoemaking, joinery, gardening, farming, work in the training shop, including picking hair, etc., mat, brush and basket making; and miscellaneous occupations, including laundry work, work in the kitchen and dairy, and other occupations of a like nature. The occupation of the girls is almost wholly in the line of sewing, knitting, and the ordinary domestic occupations. While passing through the building, Dr. Shuttleworth called my attention to a young man, then employed as an assistant to the storekeeper, who illustrated in a marked degree one of the phases of abnormal or imperfect mental development. On giving him any date of any month for a wide range of years, he would instantly and correctly tell the day of the week upon which it fell. I was informed that as his other faculties were being brought out by training and education, this peculiarity was less pronounced.

The medical care taken of the patients in this institution is in marked contrast with what I had observed at home. Instead of degenerating into a mere school, it combines in the best sense an admirable hospital, scientifically administered, with an excellent training and educational establishment. Cases are carefully recorded in well kept case-books, and the material thus accumulated is utilized for the purpose of scientific investigation.

Dr. Shuttleworth appeared fully alive to all that was going on in his special line of duty and practice, and in his last report refers briefly to some of the work which he and his assistant have done toward contributing to the general stock of medical knowledge. American alienists in visiting England ought, by all means, to visit this admirable institution.

The Morningside institution, the Royal Edinburgh Asylum for the insane was next visited. As already stated, Dr. Clouston, the superintendent, whom I had the pleasure of meeting in America, was absent on his holiday, and my view of the institution was somewhat limited. This asylum receives both private and public patients. For the latter its charges are £30 per year, and this is also the lowest rate charged for private or pay patients. Those paying this rate are lodged and fed with the paupers, but I believe have certain privileges not granted to that class. The next higher rate for private patients is £45 per year, and from this point the rates extend up to £300, with special attendants, private apartments, etc., etc.

The buildings at Morningside are the East House and West House, which are the main establishments respectively for private and public patients, and Craig House and Myrside Cottage, which are both for the private class.

I saw some portions of East and West Houses; the other buildings I did not visit. Entrance to the grounds of Morningside, which through natural and artificial means are quite inviting, is gained only through carefully guarded gates at the Lodge. The entire estate is surrounded by a wall which, though not unscalable, would make sudden elopements difficult.

West House affords accommodation for about 700 patients. Its general plan and construction, with the exception of the preponderance of dormitories over single rooms, does not differ essentially from thc ordinary American asylums. In the portions of the institutions which have not been renovated, I observed that the window panes were of small size, and that the entire sash, which was immovable, was of iron. Ingress and egress of air through the window was obtained by leaving the upper half of this iron sash unglazed, and placing outside of it an ordinary wooden glazed sash, which could be lowered or raised as desired. In other portions, more especially the day rooms and dormitories, where patients were constantly under observation, ordinary sash were used, but these only opened a few inches. Meals are served the pauper patients in a large, common dining hall.

I went about the grounds and shops, and saw patients employed in various ways. My attention was attracted to a group of a dozen patients dragging a lawn roller or mower, and in other ways I saw patients occupied at various tasks, many of them to be sure simple, but at the same time giving them the beneficial influences desired. Among others I saw "Joe," the tin-smith, described as a dangerous man, with active delusions. He was busily hammering away at his bench, and in actions and appearance carried me back to a man I had left at home employed with the engineer at the asylum, and from the necessities of the case, frequently, almost wholly, free from custodial supervision. About the grounds I observed men and women patients associating freely together, some of whom were manifestly of the "excited" class, an arrangement which appeared to me to have many objectionable features.

Dr. Clouston has a strong belief in the beneficial effects of outdoor air and exercise, and I saw very few patients in the house, no fewer, however, than I had been accustomed to seeing at home in pleasant summer weather, but the general proportion of those who spend long periods out of doors during the year, is, I doubt not, larger than at home. He takes great pains in th training of his attendants, endeavoring to carry out the views which he has enunciated in his reports and in the *Journal of Mental Science.* He has established an infirmary ward, which in future is to be the probationary ward and training-school for all new female attendants. They are to be sent there for a time at first, to begin their work by learning to nurse the sick, and to look upon all mentally-affected patients as really sick.

The apartments for private patients at East House are in many respects very elaborate, and resemble more an elegant family mansion in some of their appointments, than an asylum. The parlors, corridors and dining-rooms are handsomely decorated and furnished, and some of the suites of rooms for those who pay for such accommodations are quite luxurious.

Restraint is not employed at Morningside, nor is seclusion as often used as in some British asylums, though "padded" and "strong" rooms are provided. The shower bath, I was informed, was occasionally used in certain cases of excitement and as a remedial measure.

After Morningside, Woodilee Asylum at Lenzie was visited, and here I passed three delightful days.

When it did not rain there was a strong suspicion that the weather outside was a "wee bit saft"; but inside, Dr. Rutherford and his delightful family, together with his novelties in asylum management, combined to make the time pass all too quickly.

To write anything new in description of this now famous institution would be almost impossible, yet its management has attracted so much attention and given rise to so much discussion a to the merits or demerits of the so-called "open-door" system, that the risk of repetition can safely be incurred.

This is entirely a pauper establishment, and situated within a few miles of Glasgow, receives its patients from a portion of that active Scotch metropolis.

The asylum building, which is the most expensively constructed of the public asylums of Scotland, cost in the neighborhood of £150,000, and affords accommodation for 500 patients. It is situated about three-fourths of a mile from the village of Lenzie, upon a rolling farm of 400 acres, and commands a wide expanse of very interesting Scotch scenery. The asylum is but two stories in height, and the upper floor is used only at night—the day-rooms, sewing-rooms, etc., being all on the first floor. Of the general working of the institution I can, perhaps, give no better idea, than by quoting from Dr. Rutherford's report for 1880:

"The patients and attendants rise at half-past five. All are house cleaners until the breakfast hour, which is half-past seven. At half-past eight all go to chapel, where morning prayers are read. At nine o'clock the various working parties are arranged and inspected by medical officers, after which they go to work. At one o'clock all return to dinner. The patients and attendants all assemble in the dining hall, which accommodates upwards of 500 persons. The patients are first served, and occupy twenty minutes in taking dinner. The attendants then dine at separate tables in the same hall, the patients remaining seated during the twenty minutes allowed for the attendants' dinner. At two o'clock all leave the hall, and after having been drawn up in line and again inspected by the medical officers, resume their work as in the morning. At six o'clock all return to tea, which is served in the same order as dinner. The indoor amusements are held in the evenings—the principal of which are the weekly dance on Monday evenings, and the Wednesday evening lecture by one of the chaplains.

"On these and other similar occasions about three fourths of the patients of both sexes are present."

I arrived at Lenzie on Saturday, the afternoon of which day is considered at the asylum as a holiday. The men were engaged in cleaning and blacking their shoes, and the women in attending to the needs of their own wardrobes preparatory to Sunday, or were out on the grounds under the care of the attendants.

Services on Sunday were held in the asylum chapel, a very tastefully constructed building used solely for this purpose. Access to it is gained from the wards by glass covered passage ways, which serve at the same time for conservatories and pleasant promenades in wet weather. One of these corridors runs from the men's and one from the women's side of the asylum.

The work done by the patients can, perhaps, be best illustrated by condensing the daily returns made by the head attendant of the four divisions for each sex for one of the days I visited the asylum.

The employments enumerated for men are: At cleaning and other household work in the wards only, as garden laborers, as farm servants, as ground or field laborers, as clerks or storekeepers, as messengers, as stokers or engineers, as bakers, as tailors or upholsterers, as shoemakers, painters, joiners, plumbers or blacksmiths, masons, quarrymen, and masons' laborers. For women, occupation was furnished in the kitchen, the laundry, officers' quarters, at needle and house work, and at knitting.

The four divisions for men had on the day referred to, 45, 48, 68 and 67 patients respectively—a total of 228. The patients employed from these divisions numbered in order, 34, 15, 61 and 63—a total of 173, or about 76 per cent. of the whole number of men patients. Of these, 87 were employed as ground or field laborers, 26 as farm servants, 14 at cleaning and other household work in the wards, 13 as quarrymen, 11 in the garden, 3 as mason's laborers, 3 in assisting the night attendants, 2 as clerks or storekeepers, 2 as stokers, 2 each as tailors, bakers, shoemakers, painters and plumbers, 1 as a mason, and 1 as a messenger.

Of women, there were present on the same day in the four divisions, 44, 59, 72, and 73 patients—a total of 248. Of these there were employed on the corresponding divisions, 40, 37, 64 and 70 patients—a total of 201, a little over 85 per cent. of the women resident. Of these, 13 were occupied in the kitchen, 39 in the laundry, 3 in the officers' quarters, 101 at needle work, 52 at knitting, and 3 assisting the night attendants.

Of course it will be readily understood that of this large amount of occupation but a small proportion relatively is active, remunerative labor. Labor for the insane, however, is not what is desired, while occupation is to be constantly aimed at, and Dr. Rutherfoord seems to have attained this desideratum to a marked degree.

I saw many patients "employed" or "occupied" who were practically doing nothing; but, on the other hand, in shops and sewing-rooms, and especially in the laundry, among the women good, honest work was being performed by apparently willing hands. One method of employment I considered unique—the utilization of patients as night nurses. Six patients, three of each sex, are thus employed. They accompany the night attendants on their rounds, assist them in taking up patients when necessary, and in the general care of the sick and feble, as well as watching the epileptic and suicidal. I went through the wards at night, both with Dr. Rutherford and his assistant, and assured myself by personal inspection of the fidelity of these "patient-nurses." They, of course, only act under the supervision of the regular night attendants, of whom there are an equal number, three for each sex. The patients are encouraged to engage in this work by the allowance of a slight sum, five shillings, I believe, a month, and certain extra privileges are accorded them. On this point of the reward of patients for work, Dr. Rutherford, in the report quoted, says:

"Although I do not advocate the indiscriminate payment of patients in an asylum for their work, and deprecate the practice of giving beer, tobacco, snuff and extra food, and such like, as reward, and withholding them as punishment—for the food of a patient, including his tobacco, should be given to him in consideration of his condition and not according to the work he does, and should not require to be increased nor be allowed to be diminished; yet there are cases whose circumstances render it only just and politic that some remuneration snould be given. At present there are several patients of both sexes who receive sums varying from 5s to 20s per month. * * * * The principle is very generally admitted by the giving of beer, tobacco, picnics, and such like, which I think a wrong practice. * * * * The whole question, however, of the remuneration of patients in an asylum is a difficult one, and may be regarded as still unsolved. It is a question which has long received, and is still receiving, my careful consideration."

In addition to the number of patients accommodated in the asylum proper, some twenty men are received in the farm house at Muckroft, a portion of the farm, where they reside under the care of an attendant and his wife. They have a part of the farm and some cattle and sheep to care for. A few patients also reside at the porter's lodge, and when I was at the asylum, farm buildings were in progress of erection, in which from 40 to 60 additional patients were to be accommodated. These small communities have no connection with the asylum proper, except in drawing supplies therefrom, and being subject to its rules and regulations. When I visited the Muckroft colony on Sunday afternoon, I was received with evident pleasure, as a stranger from a far-off land, and the genial attendant in charge and the patients residing with him took evident pride in showing me over their portion of the estate. One large room was used as a sitting and dining room, another as a kitchen, and the rest of the house, differing in no way from an ordinary dwelling, was used for sleeping apartments.

In the new building in process of construction, the sleeping apartments are almost wholly dormitories, and occupy the second floor of a fine stone building, the first floor of which, together with other adjacent structures, will, when completed, be used as store-rooms, stables, vegetable-houses, etc. Dr. Rutherford has denominated this system of distributing patients about the asylum estate, "the system of location."

The attendants at Woodilee are largely Highland men and women, a race noted for their efficiency and faithfulness. They all wear a simple but distinctive uniform. That of the women is a neat, plain, black dress, white apron, white collar and a simple white nurse's cap. They impressed me as a carefully selected, intelligent and appreciative body, and to them, of necessity, belongs much of the credit for the success which has attended Dr. Rutherford's endeavors.

I now come to a subject which I approach with some hesitancy, in describing this institution, viz.—the question of locked or unlocked doors. I went about the asylum at will during my stay, and only twice, and then at night, was a key used anywhere. Entrance to all the rooms, single and associate dormitories, is gained by turning a simple handle, but these doors once shut cannot, in most instances, be opened from the inside, there being no knob, especially in the single rooms. Outer doors open and shut freely from both sides, but I observed that a patient in one of the sitting or day rooms who attempted to leave, was met at the door by the vigilant attendant, who did not resign his or her charge of the patient, until the responsibility had been turned over to some other attendant, either upon the lawn or in one of the shops or numerous working parties. Of course in individual cases who could be trusted anywhere, as in all asylums, this rule met its exceptions. There appeared to me, however, to be at Lenzie a substitution of personal vigilance for locks and keys. From nine at night till six in the morning all the outer doors are locked as much as in any asylum, and so are all the single rooms. Of the dormitories my notes do not speak, but I saw some of them under the constant watch of the night attendants. Whether the removal of apparent prevention to egress and ingress has any calming and soothing effect upon the insane, I am unable to state, and it is not the intention or province of the present article to discuss that point. Of one thing I am satisfied: it has its beneficial effect upon the attendants. It makes them realize that their intelligent watchfulness is being substituted for locks and keys, and the history of the experiment thus far at Lenzie, seems to show that as far as it has resulted in training a staff of active, vigilant attendants, it has been a success.

Notwithstanding the great apparent freedom granted the patients at Lenzie, there are arrangements for confining the violent and excited. The "strong" single rooms have double doors and the windows are guarded by inside tight wooden shutters, which slide up from the bottom and effectually prevent escape. These inside shutters are provided in many, if not all, the single rooms, and when in use offer a greater resistance to a determined effort to escape than any exterior window guard I have ever seen in an American asylum. The proportion of single rooms is about one to every five patients. Dr. Rutherford informed me, however, that he could easily do with less. With the exception of a bath-room in each infirmary, there is but one bath-room for each sex. These are large well lighted rooms, which contain a number of large earthen, porcelain-lined tubs placed side by side, a convenient distance apart, and to these bath-rooms all patients who are able are taken at least once a week. As has been before stated the dining hall is common to all patients. Only the sick and paralytic, with such as are occasionally too disturbed to dine with others, take their meals elsewhere.

The hall, which is large and pleasant, immediately adjoins the kitchen, and this allows the food to be placed on the tables while hot. I saw the patients at various meals, and never saw any disorder beyond what might arise from the congregation for a similar purpose of five hundred sane people, except in one instance. Then the offender, a talkative, mischievous girl, was quickly and quietly removed. The dietlist is not as varied as in most American asylums, but the food was well cooked and wholesome, and there was plenty of it. As would be expected in Scotland, each patient is allowed a full supply of porridge.

Visits of friends to patients are allowed but once a week, except in cases of emergency, and only on written permission of one of the medical officers.

Something will be expected, after these extended remarks upon Woodilee Asylum, upon the medical treatment there pursued. Upon this point, however, there is but little to be said. Dr. Rutherford is evidently a strong believer in the *vis medicatrix naturæ.* Only a small proportion of his patients take sleeping draughts at night, and stimulants are very sparingly employed, except in a few cases, for a short time after admission. Insanity he regards as a disease of diminished vitality, demanding invigorating treatment, for which nothing is more desirable than active outdoor employment and an abundance of fresh air.

From Lenzie I proceeded to Glasgow, and visited the Glasgow Royal Asylum, Gartnavel. I was most hospitably received by the Superintendent, Dr. Yellowlees, who, at considerable personal inconvenience, showed me everything of interest in the institution. This Asylum, like Morningside, receives both public and private patients. It was at once amusing and suggestive to observe the difference of opinion between two Superintendents, whose pauper patients at least were drawn from the same class, that existed between Drs. Yellowlees and Rutherford. The difference, however, is wholly amicable—indeed, Dr. Yellowlees seemed quite proud of the achievements of his neighbor at Lenzie, though not feeling himself justified in following his example, while Dr. Rutherford could not consent to my leaving Scotland without seeing Dr. Yellowlees.

This asylum affords accommodation for about 500 patients. Of this number, a little less than half are private patients, the excess of public patients over the private being about thirty. The general arrangements for public patients differ in no essential respect from those in America, except that the separate dining-rooms for each ward are replaced by one large dining-hall, as is quite general.

Dr. Yellowlees employs seclusion, but from the records it would appear to be used rarely, and restraint less often, though employed when, in his judgment, considered necessary. Many of the patients are on parole within the grounds, and a few for limited distances outside. Those to whom this privilege cannot be extended are placed in handsomely arranged airing courts, so situated on a gentle slope as to command a wide expanse of scenery.

Employment is furnished the patients as far as possible, and dances, concerts and other amusements are freely indulged in.

The apartments for private patients are very nicely arranged, but are not as elaborate as at some other institutions, it being the intention of the directors to provide treatment and an asylum for the insane of the middle class, who cannot afford to avail themselves of the advantages of the more expensive private asylums, and at the same time do not wish to be classified among the paupers. For those able to pay increased rates, however, the accommodations provided are in every way satisfactory.

Of this association in one institution, of the upper, middle and lower classes, the Scotch Commissioners of Lunacy speak in terms of commendation, and remark that "it is difficult to see how such a mixture of classes can be productive of anything but benefits," and say that it would be easy to give "striking examples" of this.

The increased income derived from the upper classes, over and above the actual expenditures from these patients, is used to improve the provisions for and care of the lower classes, and the whole character of the hospital is elevated, and the Commissioners of Lunacy are able to report in commendation of this plan, that "such patients" (speaking of the middle and lower classes) "enjoy advantages in the asylum much beyond anything represented by the rate of board paid for them."

I was much gratified to see here in practical operation, and receiving such unqualified approval, a system to which I was accustomed at home, and which had resulted in raising the standard of care for public patients, without in any essential way encroaching upon the rights of those who paid their own expenses.

I cannot close my notes on this institution, which, I regret, owing to the brevity of my visit, are not more full, without referring to one incident as illnstrative of the care for apparently small things exhibited by Dr. Yellowlees, when looking after the interests of his patients.

Standing in his beautiful dining hall, I asked how and where the attendants took their meals, and was informed that they ate before the patients, in a hall of their own. The object of this, Dr. Yellowlees said, was to have them in a better condition to patiently supply the patients' wants, on the theory that they would exhibit less irritability of temper, and hurry the patients less, if their meals came before, rather than after the patients'.

After leaving Gartnavel, my next visit to an asylum was at the one so well known by its published "Medical Reports," the the asylum for the West Riding, of Yorkshire, at Wakefield.

At Wakefield I was very pleasantly received by Dr. Major and his assistant, Dr. W. Bevan Lewis. The asylum building is old and in many respects unsuited for its present uses. It has, however, been pretty thoroughly renovated, and by interior decoration and changes made into quite a comfortable institution. Many of the old stone floors still remain, especially on the first floor, but as fast as possible, these are being replaced by wood. The single rooms are small, and the corridors or galleries, as they are often termed in England, are low and poorly lighted. As the patients, however, are congregated during the day, in large and well-lighted day-rooms, when not out-of-doors or employed in the numerous shops and work-rooms, the deficiency of light in the corridors is felt but little. A large proportion of the patients at West Riding Asylum are actively employed. Besides the work on the farm and the general work incident to all asylums, all the boots for patients and attendants, all the patients' clothes and most of the attendants', as well as the sheets for the patients' beds, are made and repaired on the premises. Not only is the cloth for the sheets woven at the asylum, and almost wholly by patients' labor, but at least two other varieties of cloth are made for use in the institution. I visited most of the shops and saw the patients there employed actively at work.

As would be expected by those who have read the admirable series of "Medical Reports" emanating from this institution, much attention is here paid to clinical work and pathological research. Besides the regular medical staff, there are resident at the asylum two or more clinical assistants or internes, who take much of the duty of recording clinical observations and making notes. Dr. W. Bevan Lewis, the senior assistant, had, at the time of my visit, just published a work under the title, "The Human Brain, Histological and Coarse Methods of Research, A Manual for Students and Asylum Officers," which I am glad to observe is receiving the favorable attention which it merits. Dr. Major took me through the well arranged pathological laboratory and photographing room, and showed me some very interesting preparations. It was in this laboratory, I was informed, that Ferrier's earlier studies for his writing on cerebral localization were made, and from its precincts have gone forth other no less valuable contributions to medical science.

Dr. Major uses very little restraint, but does not hesitate to apply it when, in his judgment, it is for the patient's best interests, and this ground, indeed, I found to be the one taken by nearly all the medical men in asylums with whom I conversed. They were very far from the dogmatic standpoint which some have described them as taking. A few, to be sure, could hardly conceive a case in which it was necessary, but even they admitted that when that case arose they would apply restraint with the same freedom that they would administer medicine. No candid person will deny that restraint is not employed to a greater extent in most American asylums than in those in Great Britain, but an examination of the reports of British institutions or a brief conversation with their superintendents, will soon convince any person of the fallacy of the statement which is so often made on this side of the Atlantic, that it is never used. The forms employed are, usually, the wet or dry pack, closed or endless sleeves (practically a camisole) and mittens. There appears to be a distrust among some, lest from the presence of means of restraint, its frequent use would necessarily follow. As restraint should never be applied except by direction of the physician, such distrust ought to be groundless.

I was told at Morningside that in a recent case a covered bed had been used. Concerning the case for which it was employed, and the subsequent disposition of the bed, I quote as follows from the Commissioners' report :

A large proportion of the entries [of restraint] refer, however, to the case of a male patient suffering from general paralysis, whose legs had become so much swollen and ulcerated, owing to his maintaing the erect position almost constantly both day and night. All the modes of treatment resorted to, failed to overcome this injurious habit, until he was placed in a modified form of what has been called the conservative or box-bed, in which the patient is compelled to submit to the recumbent position. The adoption of this form of restraint was so far justified by the swelling and ulceration of the legs having been cured, and the patient's general health having improved during the time it was employed. It is, however, worthy of consideration, whether the same results might not have been obtained by the adoption of means less suggestive of a return to modes of treatment which have gone out of use ; and it is recorded with approval that the exceptional nature of the case was so fully recognised by Dr. Clouston, that the bed was broken up as soon as it ceased to be used for the special purpose for which it had been constructed.

This digression is not particularly applicable to West Riding, but was suggested by the candor and freedom from dogmatism with which Dr. Major discussed this and kindred topics.

The arrangements for bathing at this asylum include a well ordered Turkish bath.

Dr. Major called my attention to gas brackets outside the transom lights over the single room doors. These are kept burning at night, in many instances, and experience, he said, had proven their utility. Many patients getting out of bed in the dark were unable to find their way back, and either stumbled about for a long time or lie down on the floor, but with the dim light burning over the door they could easily return to their beds. A fire brigade of patients and attendants has been organized at the asylum. All the patients belonging to this brigade sleep in one dormitory on the first floor, so as to ready for immediate service.

I observed among the patients under Dr. Major's care several idiots, many of whom were apparently quite young. The assosociation of this class of patients with the insane must be productive of injurious results to both. Several of the patients take exercise in airing courts, but the majority have more extended range about the estate. I went to Wakefield with very favorable impressions of the West Riding asylum, and I came away with those impressions in no wise diminished.

Proceeding from Wakefield to London I was at a convenient point to reach several institutions. My time, however, only permitted visits to Bethlem, Hanwell, Colney Hatch and Brookwood.

At Bethlem I fortunately found Dr. Savage at home, and he very kindly escorted me about the hospital explaining its peculiar features.

All poor lunatics presumed to be curable are eligible for admission to Bethlem for maintenance and medical treatment. Patients who have sufficient for their maintenance in a private asylum, those who have been insane more than a year and are considered incurable, idiots and epileptics, those whose condition indicates speedy disolution of life, and those who require special attendance are excluded. A preference is also given to patients of the educated classes, and to ensure accommodation for these, patients who are proper subjects for a county lunatic asylum, are not received.

The present building has been occupied since 1815, and is not, therefore, constructed upon modern plans. In some respects its exterior reminds me of the State Lunatic Asylum at Utica, and some of the interior arrangements are not unlike those of the latter institution. The wards are pleasant, and by pictures and flowers are made as home-like as possible. In addition to the hospital proper, there is a house at Whitely for convalescents, where about thirty patients are received and enjoy an agreable change from the monotony of asylum life. Patients are sent out to this branch for varying periods of time, from early spring till the opening of winter, and the change is said to be productive of much good.

The patients at Bethlem have large and well arranged airing courts for exercise, and in their use the disturbed or excited patients are separated from the more quiet and orderly. Padded rooms and seclusion are employed, but restraint is rarely used.

Medical treatment is more systematically carried out at Bethlem than at many British asylums, and pathological investigations are regularly pursued.

Dr. Savage lectures at Guy's hospital on psychological medicine, and also gives clinics, to classes of ten each at Bethlem. The students forming these classes are brought directly into coniact with the patients, instructed in making certificates, are made to examine patients and to diagnose their form of insanity, and make a written report, and subsequently are examined, upon a special case assigned to them.

The attendants at Bethlem are not uniformed. They impressed me as intelligent and well trained. Two clinical assistants aid the regular medical staff in the care of the patients.

One point which has so much been insisted upon in the pages of this journal, the necessity of early treatment, is strikingly illustrated by the statistics of Bethlem. As has already been stated, patients who have been insane over a year are not received, unless thought curable by the medical officers of the asylum. Of the 269 admissions for 1881, but seven were insane over eleven months. The recoveries for the same period were 141, over fifty per cent. of the admissions, and of these none had been insane over ten months, and but twenty-seven had an insane history of more than three months when admitted.

Dr. Savage allows many of his patients an extended parole, sending them freely into the streets of London without an attendant, even when their mental disturbance is still quite manifest. He informed me that he had met with but little trouble in granting his patients paroles of this character. He cited the case of one woman who made some objectionable acquaintances, and said a few patients had refused to return, but no serious difficulties had arisen.

Hanwell, the scene of Conolly's labors, now affords accommodation for 1,800 patients, 700 men and 1,100 women. As would be expected from the traditions connected with this institution, restraint is seldom, if ever, used. Seclusion is, however, employed, and I saw a number of "strong" and "padded" rooms. A large proportion of the patients are empleyed in shops and about the premises, and the women, many of them, do sewing and knitting, and are employed in general domestic work. I saw, in passing through the wards, a large number of aged and infirm patients, as well as many epileptics and paralytics. The superintendence of the men's division devolves upon Dr. Rayner, and that of the women upon Dr. Richards, and to the latter gentleman I am indebted for my knowledge of the institution. The major portion of the asylum is old, and the wards impressed me as dark and gloomy. An attempt, however, has been made to relieve this, by stenciling the walls in bright colors.

The arrangements for night supervision are practically the same as in most of the asylums I visited. In the large dormitories for suicidal and epileptic patients, I observed at one end what, indeed, I had noticed in other asylums—small rooms partitioned off by ordinary matched boards. These board partitions only extend to the height of eight feet from the floor, and the tops are left open. These little apartments are called "cubicals," from the fact, I presume, that their interior dimensions, measuring from the top of the partition, form cubes of eight feet. In these are placed any patients who, during the night, cannot be properly cared for among the other patients in the dormitories, but who at the same time demand continuous supervision. These arrangements seem to have met with general favor, and have, I understand, received the commendation of the Lunacy Commissioners. A new and very handsome chapel has been recently built at this asylum, at a cost of several thousand pounds.

I saw no actively disturbed patients, and those who were apparently the brightest did not impress me favorably. Indeed, the general air of mental deterioration which pervaded the wards of this asylum and Colney Hatch, as well as to a greater or less extent most of the asylums I visited, was painful. I saw very little of the brightness and intelligence which is observable in the convalescent wards of American asylums. In this respect Bethlem more nearly represented what I had been accustomed to seeing at home than any institution exclusively for public patients that I saw in Great Britain.

At Colney Hatch, Dr. Marshall, medical superintendent of the women's division, very kindly conducted me over that extensive asylum. The patients at this asylum, as at Hanwell, come from London, and are all paupers.

The interior arrangements do not differ from that of the other institutions described. There are few single rooms, dormitory sleeping accommodation preponderating. "Padded" and "strong" rooms are employed for purposes of seclusion, but restraint is seldom used. Not as large a proportion of patients are reported as employed in this asylum as in some I visited, but this is in a measure accounted for by the character of the patients, drawn from the idle and dissipated classes of London, and by the large number of paretics under care. At the time of my visit, some of the wards were undergoing renovation, and new buildings were in process of construction, upon which the work of patients was being utilized, especially in moving bricks, etc.

Of my brief visit at Brookwood my notes make little mention. I came away, however, with very pleasant impressions of the institution, and especially was I pleased with the new block which had recently been opened. Dr. Barton, the new superintendent of this asylum, was formerly an assistant under Dr. Brushfield, who had a short time previously retired from that position, and appeared to be maintaining the efficient management of his well known predecessor.

The Lancashire Asylum, at Prestwich, was the last one I had the pleasure of visiting. I had been strongly advised to make a point of visiting this asylum, and seeing some of the very fine decorative effects which had been produced under Dr. Ley's direction.

I arrived at Prestwich at a somewhat unfortunate time, as the medical superintendent, Dr. Ley, and his assistants, were engaged in giving testimony to a Coroner, who was inquiring into the death of a patient, killed a day or two previously by another patient in the asylum. As soon as possible, however, Dr. Ley closed his testimony, and very kindly conducted me about the buildings and grounds.

The circumstances of the homicide above referred to were as follows: A patient, sweeping one of the wards, was bothered by another getting in his way, and pushed him aside. The annoyance continuing, he struck him on the head with the broom handle and knocked him down, causing either by the blow or fall a fracture of the skull, resulting in a few hours in death. The man committing the assault was not considered dangerous or violent, and on subsequent examination did not appear to have had any motive in striking his fellow patient, beyond driving him out of his way. I was informed that he would be taken before the next assizes, and sent to Broadmoor, the criminal asylum.

Dr. Ley related an incident connected with the unfortunate occurrence, which pointed to the fact that a certain style of sensational irresponsible journalism had an existence in England as well as America. He had offended a young and ambitious newspaper reporter, a few months before, by refusing to give him any data upon which to write a sensational newspaper article concerning the asylum. In order, therefore, to have his revenge, he prefaced his account of the homicide with glaring head lines, "Another murder in the Prestwich Asylum," "The second within a few months," etc., and then drew on his imagination for the details, describing a bloody struggle and a maniac's fury, etc., in the style which is too common in this country. He had conveniently forgotten that the other accident, to which he referred as occurring within a few months, had happened over three years ago! But sensationalism dies when facts are introduced. This incident, so closely resembling some American newspaper methods, was the only one of the kind which came to my attention while abroad, but I learned that it was not by any means the only case of malicious misrepresentation of asylum officers which had occurred, and that the evil was growing.

I soon forgot the unpleasant impressions created by the relation of the above incidents, when I was ushered into the pleasant, one could almost say delightful, wards of the Prestwich Asylum. Everywhere were light, decoration, pictures, birds and flowers. The walls were stenciled in attractive colors and forms, and artistic shelves and cabinets adorned them. The furniture, made for use, was at the same time ornamental, and the pictures, though inexpensive, were such as attracted and pleased the eye. Noticing numerous apparently bronze busts and statuettes on ornamental brackets about the walls, I asked if they were not expensive, and was informed that the asylum bought the originals, some patients made molds from these and made plaster casts, which were afterward painted in imitation of bronze. The brackets, cabinets and shelves which I so much admired were made by patients, and, as I learned from another superintendent, from designs by Dr. Ley. I saw patients veay busily stenciling a wall in pretty and tasteful patterns. Remarking that he was fortunate in possessing such skilled labor, the superintendent replied that none of the patients then at work had ever done any common painting even, before coming to the asylum. They had been there for some years, and had been educated to the work. In several wards large sections of the external wall had been cut out, columns introduced to support the roof, and large glass-roofed bays thrown out for sitting and workrooms, extending, in some instances, nearly the entire length of the ward. The large dining hall was made cheerful and inviting by potted plants and hanging baskets, and not having to fear a temperature of twenty-five degrees below zero, Dr. Ley had introduced windows in roof and wall in great profusion.

The infirmary used for paralytics and the sick and feeble, was one of the most cheerful looking wards in the building. At one end a large fire-place, with ornamental mantel and cabinet surmounting it, afforded warmth and increased ventilation, and, as in the other wards, flowers, birds, pictures, etc., were here supplied with lavish hand. Beside each bed a small table was placed with the patient's dinner, for those not able to be up, while in sunny or pleasant corners, were the less feeble, in rolling or reclining chairs of various styles, with ingeniously fashioned stands or tables to hold their food or work.

Dr. Ley rarely uses restraint, and seclusion is seldom employed. He informed me that with all his profusion of glass, pictures and other articles of ornamentation, he rarely had any breakage, even in his reception ward, where all new admissions go for a time.

Large and cheerful dormitories are used for the suicidal and epileptic, where they are under proper supervision at night, as well as those in the infirmary wards. Adjoining the main asylum building is a block, like the main building, two stories in height, occupied by about a hundred patients, mainly workers, where the "open door" system is practically employed. As would be expected from the interior, the surroundings of the asylum are made conducive to the patients' comfort. There are tennis-courts, cricket-fields, and bowling greens, while the farms and shops where various trades are carried on, afford the needful occupation to which much attention is here paid. As at the other Lancashire asylums, an annex is being erected at Prestwich. It s to hold 840 beds, and when I was at the asylum was rapidly iadvancing toward completion. The upper floor was intended solely for sleeping, the lower floor being devoted to day-rooms, sewing-rooms, and a certain number of sleeping apartments. Like the old asylum, it is to be warmed by steam, though fire-places are also to be used, largely for purposes of ventilation. The ceilings are not plastered; the joists supporting the floor are planed, and with the under surface of the floor they support, are varnished. The kitchen for the annex is a model in many respects, and immediately adjoins the large common dining hall. New gas works to supply both buildings had just been finished.

I think all who have visited Dr. Ley's well conducted institution will echo my wish that he would publish illustrated descriptions of some of the interior arrangements of his asylum.

In closing this hurried and imperfect account of a delightful visit to these asylums, I feel it a pleasant privilege to record my hearty appreciation and thanks for the many courtesies which were extended to me by the gentlemen connected with the various institutions which I visited.

I saw many things worthy of emulation; other things which I could not approve, but which, not being able to place myself in exactly the same standpoint the asylum officials occupied, I could not condemn.

I found a very fair appreciation of American institutions and American methods, and a fairer and better judgment of our conduct of asylums than, unfortunately, exists at home in certain quarters.

It may be of interest, in a future article, to glance at the differences in lunacy administration between the two countries. I cannot admit, with a recent writer, that our country surpasses the world in the arts and sciences, or that she leads in social and political advancement; nor do I believe with him that she has been wofully laggard in the treatment of the insane. The thoroughness and stability of Great Britain's political economy are reflected in her lunacy administration, but it is within the memory of Lord Shaftesbury, the illustrious head of the English Lunacy Commission, when a far different state of affairs existed; and the reforms of the Tukes, Hill, Charlesworth and Conolly are of comparatively recent date.

